# Management of Complete Cricotracheal Separation: A Case Report From a Level 1 Trauma Center

**DOI:** 10.7759/cureus.80826

**Published:** 2025-03-19

**Authors:** Madhur Uniyal, Santhosh Balachandra, Ruby Kataria

**Affiliations:** 1 Trauma Surgery and Critical Care, AII India Institute of Medical Science - Rishikesh, Rishikesh, IND

**Keywords:** airway injury, airway mangement, cricotracheal separation, laryngeal repair, laryngeal trauma

## Abstract

Complete cricotracheal separation is a rare and severe form of laryngeal trauma that most clinicians encounter infrequently, resulting in limited experience with its management. This report discusses the intricate management of a patient with complete cricotracheal separation following a traumatic incident. We present this case to highlight the challenges in the diagnosis and airway management of complete cricotracheal separation. The patient was transferred to our level 1 trauma center with a secured airway via an endotracheal tube from the referring hospital, where they experienced respiratory distress during an extubation attempt before transfer. Respiratory distress may be due to an air leak in the trachea upon deflation of the endotracheal tube cuff. Upon admission, the patient exhibited stable respiratory function, and there were no overt signs of laryngeal injury aside from subcutaneous emphysema around the neck. Imaging via a computed tomography scan revealed a distorted cricotracheal framework, raising suspicion of a high-grade airway injury and prompting surgical exploration. Upon surgical exploration of the neck, a complete cricotracheal transection was discovered with a wide separation of the tracheal ends and an inflated endotracheal tube cuff, which surprisingly prevented any air leak. Intraoperative management included performing a tracheostomy distal to the injury site, followed by a primary end-to-end anastomosis of the cricotracheal injury. This case underscores the crucial importance of prompt and efficient airway management in patients with complete cricotracheal separation. This case underscores the critical need for high clinical suspicion in subtle presentations, the importance of early surgical intervention, and the role of airway devices in stabilizing severe laryngeal trauma.

## Introduction

Cricotracheal separation is one of the most severe and life-threatening injuries to the airway [1]. Although rare, it carries high morbidity and mortality due to the risk of complete airway obstruction with a mortality rate ranging from approximately 26.8% to 40% [2]. This condition poses significant challenges in diagnosis and management due to its atypical presentation and the critical importance of prompt airway stabilization. Given its crucial role, disruption of the cricotracheal junction can lead to rapid respiratory failure if not promptly identified and managed.

In cases of complete cricotracheal separation, the trachea becomes completely detached from the cricoid cartilage, leading to a potential loss of airway continuity. This can typically result from direct neck trauma, hyperextension injuries (e.g., dashboard impact in motor vehicle collisions), or penetrating wounds [3]. Due to its infrequent occurrence, clinicians may struggle with timely recognition and optimal management strategies [4].

The presentation of cricotracheal separation can vary widely. While some patients may exhibit overt signs of airway compromise, others might present with more subtle symptoms. Common clinical features include respiratory distress, subcutaneous emphysema, hoarseness, and difficulty swallowing. In this case, the patient exhibited respiratory distress during an extubation attempt at a referring hospital but showed no obvious signs of laryngeal injury upon admission, aside from subcutaneous emphysema around the neck. This highlights the potential for atypical presentations that can complicate diagnosis.

Early and effective airway management is paramount in patients with cricotracheal separation. Delays in securing the airway can lead to rapid deterioration and severe hypoxia [5]. Imaging studies, such as computed tomography (CT) scans, are essential for assessing the extent of the injury and planning the surgical approach. The decision to perform a tracheostomy and primary end-to-end anastomosis of the cricotracheal injury must be made promptly to ensure the best possible outcome for the patient [6].

This case report aims to provide a detailed account of the management of a patient with complete cricotracheal separation at a level 1 trauma center. It emphasizes the importance of early recognition, the challenges associated with atypical presentations, and the critical nature of prompt surgical intervention. The unusual intraoperative finding of an inflated endotracheal tube cuff preventing air leaks further underscores the complexity and unique aspects of managing such rare and life-threatening injuries.

## Case presentation

A 41-year-old male presented to the trauma emergency department after sustaining injuries in a motorcycle accident, where he collided with a road divider. The patient presented with a secured airway via an endotracheal tube from the referring hospital, where he experienced respiratory distress during an extubation attempt. A left intercostal drainage tube was placed for the left pneumothorax. The patient was fully conscious and was responding to the commands. Subcutaneous emphysema was noted from the neck to the chest, though no bruising or lacerations were present on the neck. The subcutaneous emphysema in this case may also be attributed to a left pneumothorax. Neck swelling obscured the palpation of the laryngeal framework, making assessment challenging. Oxygen saturation ranged from 98% to 100% with a FiO_2_ of 35% on CPAP mode. The patient had normal pulse rate and blood pressure. 

**Figure 1:**
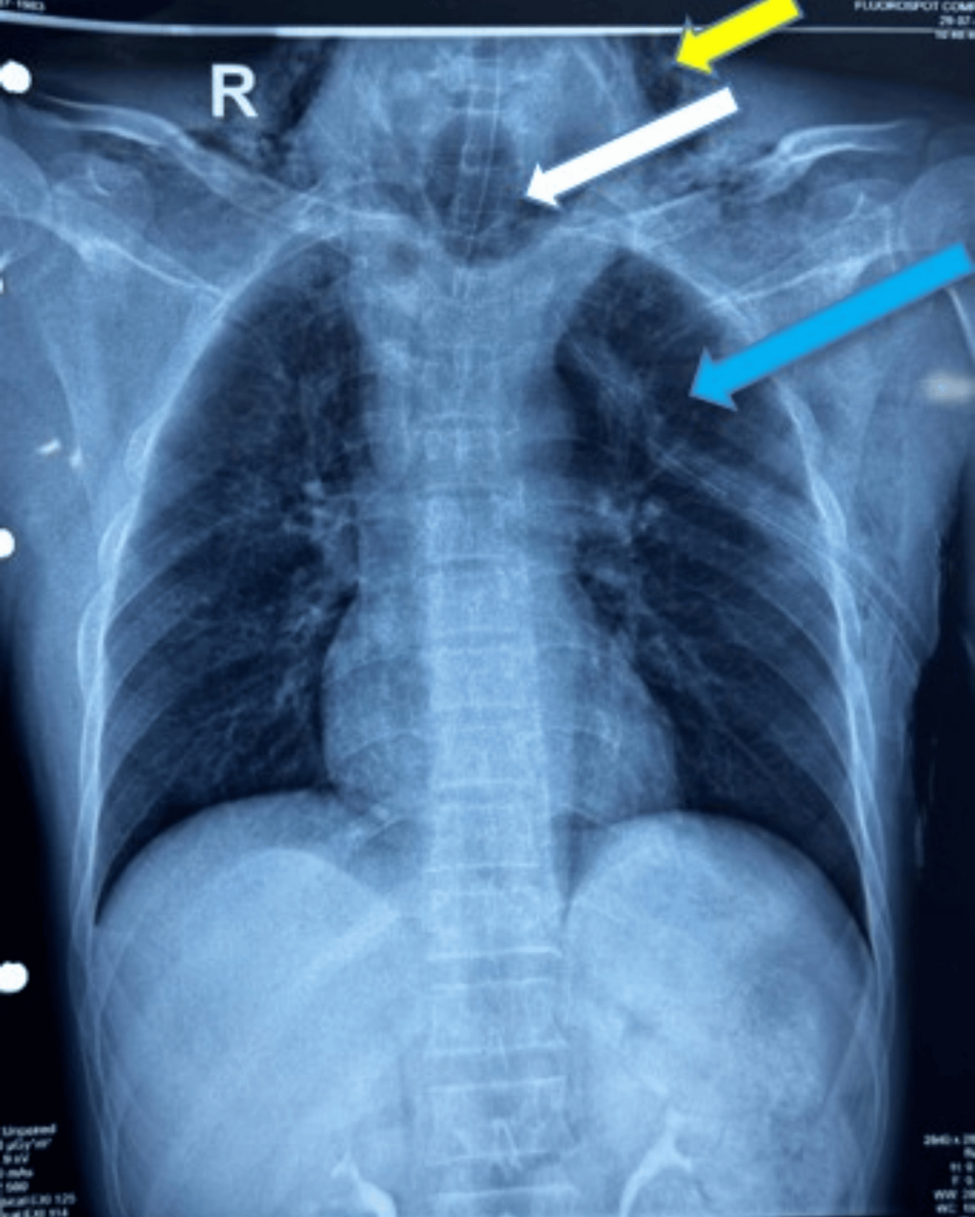
Chest X-ray revealed subcutaneous emphysema (yellow arrow), an inflated endotracheal tube cuff in the trachea (white arrow), and a left pneumothorax with an intercostal chest tube (blue arrow).

A chest X-ray showed a bilateral clavicle fracture with left pneumothorax with ICD in situ with inflated endotracheal tube balloon (Fig. 1).

**Figure 2:**
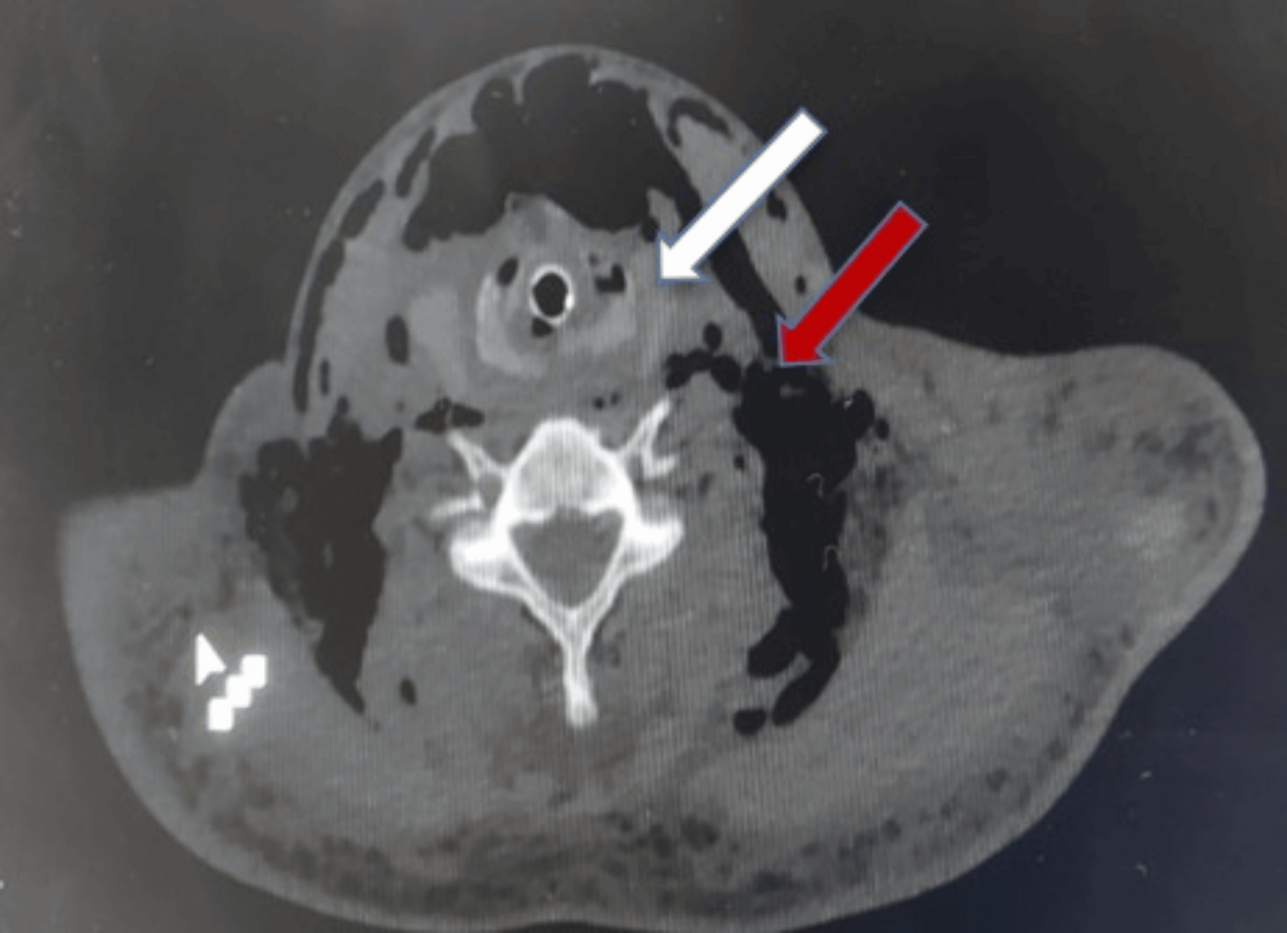
CT scan showing subcutaneous emphysema around the neck (red arrow) with distortion of laryngotracheal framework (white arrow)

A computed tomography scan of the neck and chest showed pneumomediastinum and subcutaneous emphysema around the neck and chest wall and also showed distortion of the laryngotracheal framework with an inflated endotracheal balloon. In addition, it showed a left pneumothorax, bilateral first-rib fractures, a right second-rib fracture, and bilateral mid-shaft clavicle fractures (Fig. 2). These findings raised concerns for significant airway and thoracic trauma, necessitating prompt surgical intervention.

**Figure 3:**
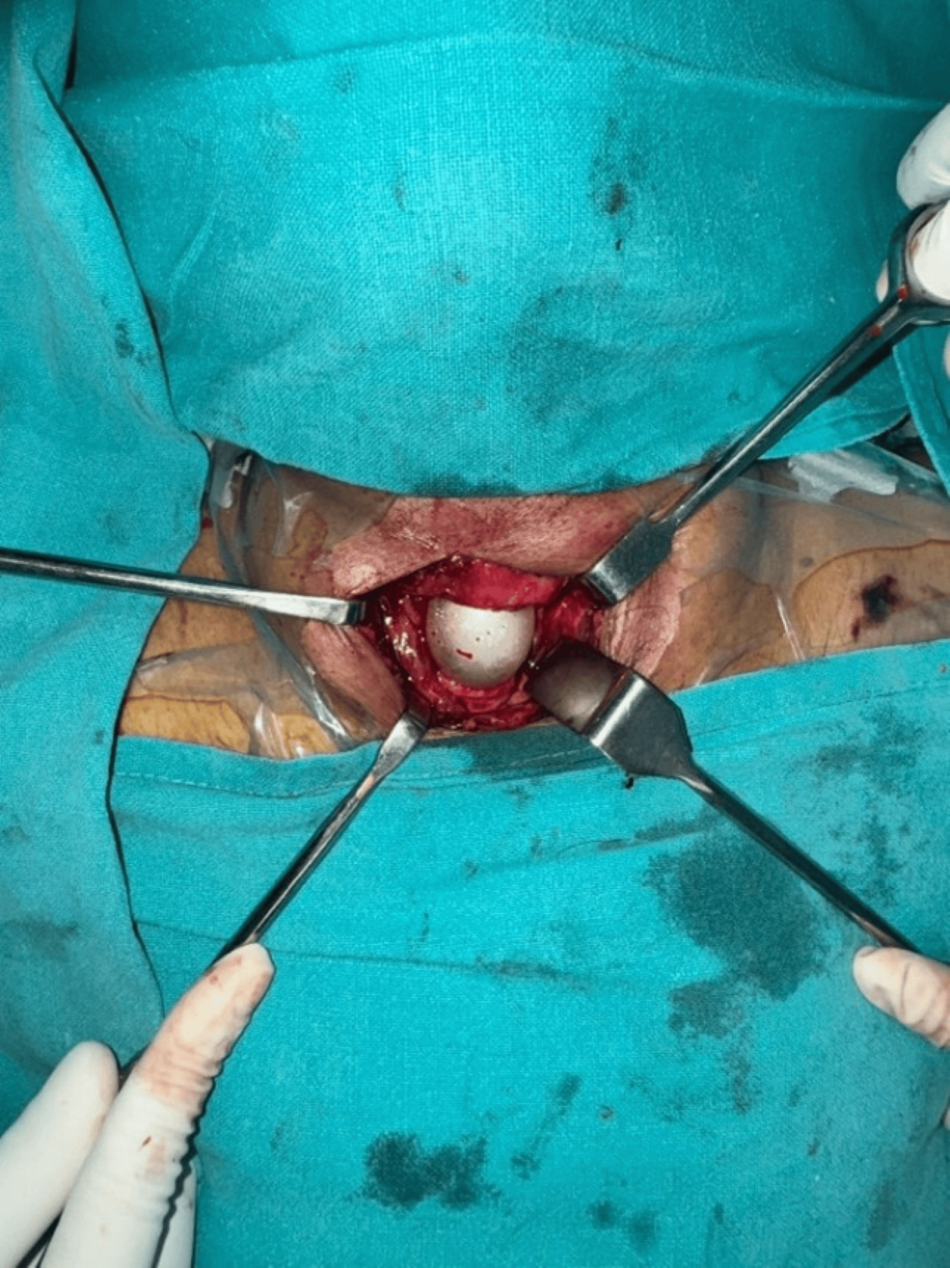
Intraoperative image of an inflated endotracheal tube balloon abutting the cricotracheal separation

The patient underwent neck exploration the same day. Intraoperatively, upon separating the strap muscles and incising the pretracheal fascia, a complete cricotracheal transection of around 4 cm was discovered with a wide separation of the tracheal ends and an inflated endotracheal tube cuff, which effectively sealed the airway and prevented any air leak (Fig. 3).

**Figure 4:**
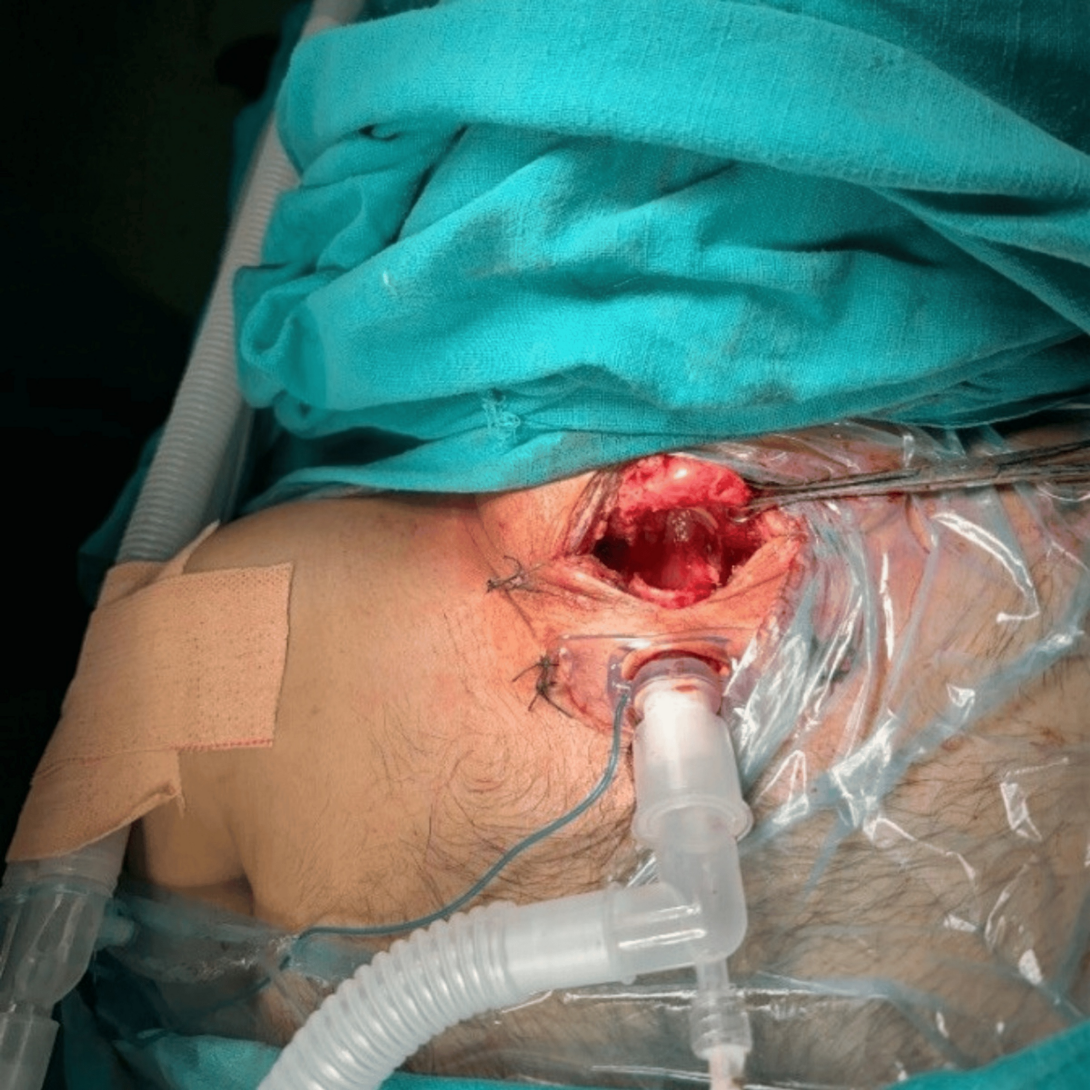
Intraoperative picture of cricotracheal separation with intact posterior wall mucosa

Upon removing an endotracheal tube, there was a complete cricotracheal transection with a wide separation of the tracheal ends (Fig. 4).

**Figure 5:**
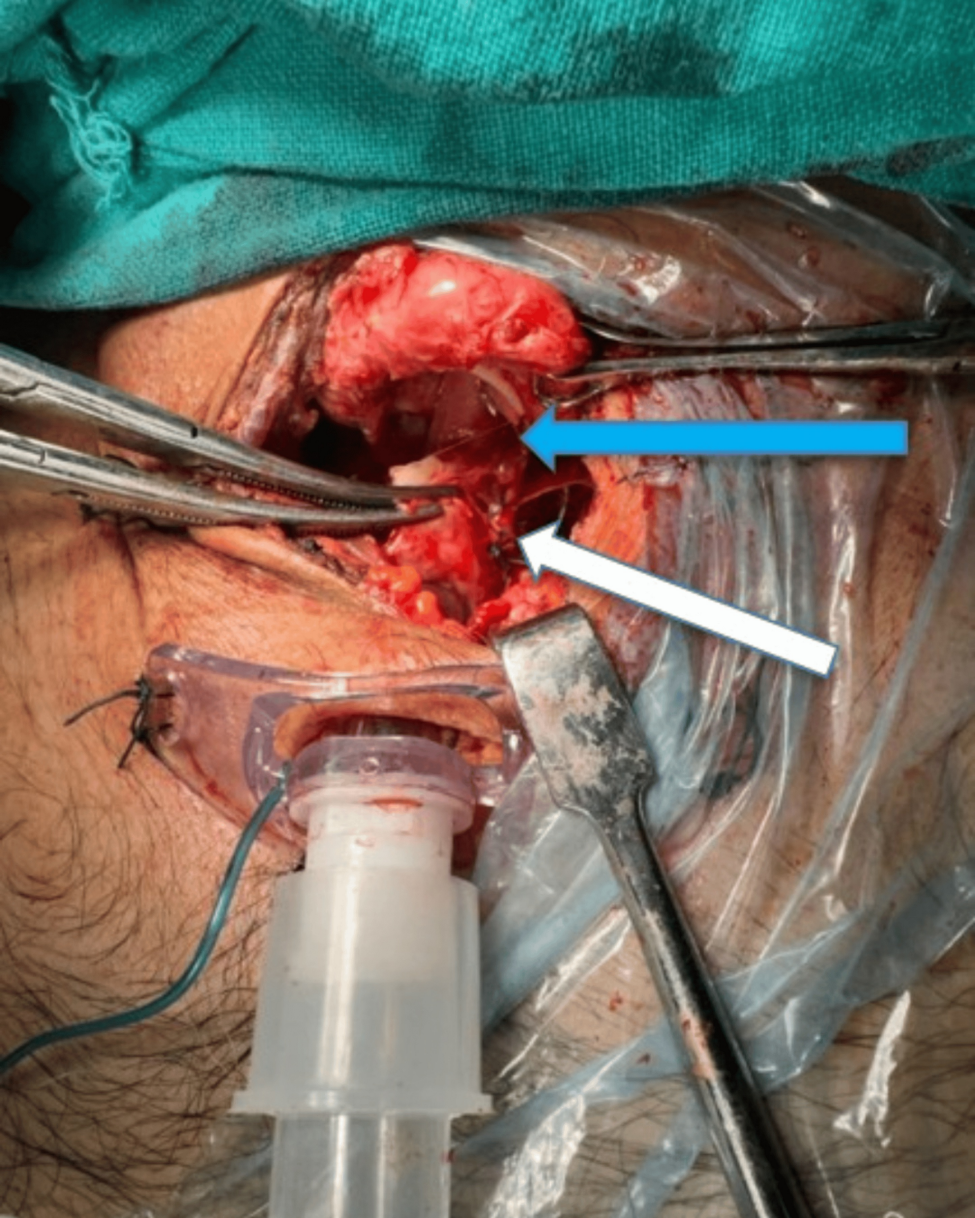
Image showing cricotracheal separation (blue arrow) with repair of a small vertical tear at postero medial aspect, distal to cricotracheal transection (white arrow)

A small vertical tear of 1 cm was noted in the left posteromedial aspect distal to the cricotracheal transection (Fig. 5).

Given the extent of the injury and the need for a secure airway, a tracheostomy was deemed the safest approach distal to the injury site, followed by a primary end-to-end anastomosis of the cricotracheal injury using interrupted sutures with a Prolene 2/0. 

Postoperatively, the patient was kept nil per oral for 24 hours, after which enteral nutrition was initiated via a nasogastric tube. Oral intake was reintroduced on day 7 following clinical assessment. Sedation was gradually reduced over one week. A direct laryngotracheoscopy confirmed a well-maintained and open cricotracheal airway with sutures intact and without granulation tissue. Movement was observed in both vocal cords. The patient showed steady improvement, successfully transitioning off the nasogastric tube without experiencing aspiration while eating or drinking. He was able to produce sound when the tracheostomy tube was occluded and displayed a strong cough reflex. He was discharged with the tracheostomy in place with a plan to follow up after two weeks for cuff deflation trials (gradual decrease in cuff inflation to assess airway patency) and downsizing the tracheostomy tube. As there were no signs of vocal cord immobility before discharge, a full recovery with proper vocal cord function is expected. However, the possibility of subglottic stenosis remains a consideration. The patient gave written informed consent for the publication of clinical details and images.

## Discussion

Laryngeal injuries are rare, accounting for just 1% of all trauma cases [7]. Laryngotracheal separation is the most severe form of laryngeal trauma. Timely and proper airway management is life-saving [8]. This case is particularly notable due to its atypical presentation and the role of the endotracheal tube cuff in preventing immediate airway compromise [9]. Tracheal injuries often present with subtle and relatively nonspecific signs and symptoms that may not correlate well with the severity of the underlying injury. In some cases, intact peritracheal tissue can act as a lifesaving conduit for gas exchange through the disrupted airway [10]. The most common clinical manifestations include respiratory distress, dyspnea, poor gas exchange, and hemoptysis [11]. Cyanosis and severe respiratory compromise are observed in 30% of cases. In addition, hoarseness or dysphonia is a frequent symptom, noted in 46% of patients [12].

Studies showed that the most frequently reported indicators of airway injury include subcutaneous emphysema (occurring in 35-85% of cases), pneumothorax (20-50%), and hemoptysis (14-25%). However, these signs are often nonspecific, and the injury may not be immediately apparent, leading to diagnostic delays. In addition, deep cervical emphysema and pneumomediastinum are present in roughly 60% of individuals with tracheobronchial injuries. A significant proportion of these injuries (25-68%) are not immediately diagnosed. Therefore, physicians must maintain a high suspicion when encountering non-specific signs such as dyspnea, subcutaneous emphysema, cough, and hemoptysis [13].

The mechanism of injury, vocal alterations, and rapidly expanding subcutaneous emphysema in the neck serve as key diagnostic indicators. A thorough clinical assessment should be complemented by radiologic imaging, angiography, CT scans, and tracheobronchioesophagoscopy [14]. Early identification of occult upper-airway injuries relies heavily on the accurate interpretation of chest radiographs. If initial radiographs fail to confirm a diagnosis, a CT scan is advised. Preoperative CT is particularly useful for detecting concurrent laryngeal or unsuspected chest injuries that might require attention during surgical intervention. However, CT is not recommended for trauma patients who are hemodynamically unstable or have an unstable airway. In this case, CT findings of laryngotracheal framework distortion prompted early surgical intervention, emphasizing its role in stable patients. Helical CT with 3D reconstruction is an effective screening tool for suspected tracheal rupture and can help determine the necessity of bronchoscopy [15]. Bronchoscopy remains the gold standard for diagnosis, with flexible bronchoscopy being the preferred initial approach to assess the location and severity of the injury.

The management principles emphasize the urgent establishment of an airway through emergency tracheostomy, prompt wound exploration, and immediate surgical reconstruction via end-to-end anastomosis, all of which were followed in this case. If the cricoid remains intact, a nonabsorbable suture should be placed from the upper cricoid to the lower aspect of the second tracheal ring, ensuring knots are tied extraluminally. This technique helps restore airway continuity while minimizing tension on the anastomosis, reducing the risk of restenosis. In cases where this approach is unfeasible due to extensive injury, thyrotracheal anastomosis may be performed. Complete tracheal transection requires meticulous suturing while taking care to avoid damage to the recurrent laryngeal nerves [16].

As noted by Holinger and Johnston [17] and Curtin et al. [18], laryngeal fractures should be managed similarly to fractures elsewhere in the body by restoring anatomical alignment and stabilizing the structures in a functional position. Surgical intervention aims to preserve the airway, minimize secondary complications during healing, and restore laryngeal function. In cases of cricotracheal separation, the standard approach includes emergency tracheostomy, neck exploration, and immediate reconstruction [19]. Consensus suggests that surgical intervention should ideally be performed within 24 hours of injury [19], although Maran et al. described “early” intervention as occurring within one week. Delayed surgical treatment is associated with poorer airway and voice outcomes. Long-term complications of laryngeal trauma may include persistent voice abnormalities, recurrent granulation tissue formation, airway stenosis, and chronic aspiration. In this case, the patient retained vocal cord mobility before discharge, suggesting a favorable prognosis, although long-term monitoring for stenosis remains essential. Long-term complications of laryngeal injuries may include the necessity for a permanent tracheostomy and vocal cord dysfunction. In a review of 414 cases of laryngotracheal trauma, Gussack et al. reported that 17% of patients experienced persistent airway difficulties, while 21% had significant voice impairment. The development of granulation tissue and airway stenosis can hinder decannulation. However, cases of delayed recovery have been documented, highlighting the importance of allowing a recovery period of at least six months to a year before considering any rehabilitative procedures [20].

## Conclusions

In summary, laryngeal injuries must be managed with utmost care as they can be life-threatening. A high degree of suspicion is warranted in any patient presenting with subcutaneous emphysema of the neck. The primary objective is to secure the airway, and the decision regarding endotracheal intubation should be made swiftly but only after careful evaluation.

This case highlights the critical role of timely and effective airway management in patients with complete cricotracheal separation. It also underscores the difficulties encountered due to the rarity of this condition and its atypical presentation without evident laryngeal injury. The intraoperative discovery of an inflated endotracheal tube cuff preventing an air leak further illustrates the complexity of such cases. This case also highlights how an inflated endotracheal tube cuff can temporarily maintain airway integrity despite complete cricotracheal separation, emphasizing the unpredictable nature of such injuries. In cases of partial airway transection, blind endotracheal intubation should always be avoided, as it may result in complete cricotracheal separation. Early surgical repair of laryngeal injuries is recommended, as it significantly improves both voice and airway outcomes. 

Long-term complications of laryngeal trauma may include persistent voice abnormalities, recurrent granulation tissue formation, airway stenosis, and chronic aspiration. In this case, the patient retained vocal cord mobility before discharge, suggesting a favorable prognosis, though long-term monitoring for stenosis remains essential.
